# The Differential Diagnosis of the Cause of Sudden Unconsciousness

**Published:** 1882-08-20

**Authors:** R. O. Beard


### THE DIFFERENTIAL DIAGNOSIS OF THE CAUSE OF SUDDEN UNCONSCIOUSNESS.

BY R. O. BEARD, M. D.

It is a matter of wonder that to a subject of such grave importance, as "The Differential Diagnosis of the Cause of Sudden Unconsciousness," so little distinctive attention has been paid, for though unconsciousness be but a *symptom* of diseased or perverted action, it is one of such vital consequence, often one of such imminent danger, that a fortunate or fatal issue frequently turns upon the pivot of the prompt diagnosis and intelligent care of the physician.

Even granting that in any given case all idea of an immediately fatal tendency is eliminated—supposing that the conditions point to nothing more serious than ordinary inebriation—it must not be forgotten that upon the doctor's diagnosis and the expression of his opinion may hinge not only his own reputation, but also the moral character of a, perhaps, hitherto innocent individual.

Mistakes of this nature have been made, even by reputable practitioners, and more than one victim has in consequence, suffered death in forced confinement, without care or treatment, and found vindication only in an autopsy which revealed an organic lesion as the cause, first of coma, and subsequently of disease.

The need of a careful study of the various causes productive of this phenomenon is thus prominent, because their differentiation is apt to be so obscure that physicians of undoubted ability have found themselves baffled.

In this paper I propose to consider only those conditions in which unconsciousness is of sudden occurrence, unattended by marked or continued prodromata, and sufficiently complete to render the patient incapable of furnishing subjective evidence or prior history. I shall endeavor to outline the distinctive symptoms observable in these cases, and as the readiest means of studying these I shall append a summarized table which may prove of some diagnostic value.

The possibility of the co-existence of two or more diseases giving rise to this condition, or of the complication of disease with accident, or *vice versa*, must always be borne in mind. Apoplexy or sunstroke may supervene in a state of acute alcoholism; cerebral congestion or embolism may be accompanied by slight hemorrhage. An apoplectic attack has been known to follow an epileptic seizure, and contusion, concussion and compression of the brain may be combined as results of severe injury.

Under the generic term of *Apoplexy*, a relic of the old-fashioned nomenclature, is grouped a class of cases almost involving coma, but with variations sufficiently distinct to indicate the differing seat and character of the causative lesion. These are, (1) cerebral congestion, (2) cerebral hemorrhage, and (3) meningeal hemorrhage. In

CEREBRAL CONGESTION

unconsciousness is rarely of primary occurrence. It is usually preceded by general hyperæmic symptoms, and only in occasional cases is the initial evidence of disturbed function. Its distinctive features are: a partial paralysis, frequently bilateral but rarely, if ever, hemiplegic, contracted pupils with feeble reaction, temperature continuously higher than normal, respiration slow and labored but lacking the stertor and peculiar expiratory puffing of the lips and cheeks observed in cerebral hemorrhage, venous distention of the face and neck, and the comparatively rapid restoration of mental and muscular power.

This condition is not unfrequently superinduced by sunstroke or alcoholic stimulation, and if prolonged may end in serous effusion and death.

The existence or non-existence of hypertrophy of the heart, as an exciting cause, should be determined, and may serve as a valuable aid in diagnosis.

The opinion held by Trousseau that so-called apoplectiform congestion is always epileptic in character is certainly untenable, not only because many of the general symptoms are essentially different, but also because an epileptic attack in which coma is as prolonged as it is in many cases of cerebral congestion must constitute the graver form of the disease and must involve convulsions so pronounced as to render mistake impossible.

CEREBRAL HEMORRHAGE, unlike the foregoing, is always marked by sudden unconsciousness. The probable age of the patient is a question valuable in diagnosis, as hemorrhage rarely occurs before forty. The generally complete suspension of intellection, sensation, voluntary and reflex motion, the occurrence of true hemiplegia, the relation or paralysis of the sphincters, the stertorous and puffing character of the respiration, the varying temperature, the inequality and insensibility of the pupils, and the frequent lateral deviation of the eyes and head toward the non-paralyzed side, are its most important characteristics.

Those rare cases in which the *Pons Varolii* is the seat of hemorrhage are most difficult of diagnosis, because the pupils are apt to be equally contracted, the respiration lacks the characteristic stertor, and paralysis is long delayed and often di-facial. This condition is, in particular, closely simulated by opium narcosis.

MENINGEAL HEMORRHAGE, although of rare occurrence, is a distinct cause of sudden and profound coma. Its symptoms differ little from those of cerebral hemorrhage.

A tendency to a remission and recurrence of the attack, the usual appearance of a general motor and sensory paralysis instead of hemiplegia, and the non-impairment of reflex action, are the only differential points we can observe, and whilst these may indicate an involvement of the meninges, it is not easy to determine whether the meningeal lesion is a primary one or whether it is consecutive to a cerebral hemorrhage by process of invasion.

CEREBRAL EMBOLISM

is another usual cause of sudden unconsciousness. Although possible at any age, yet the youth of a patient attacked should be regarded as presumptive evidence in favor of an embolus opposed to hemorrhage. The existence of endocarditis or of a valvular heart lesion also offers good grounds for a suspicion of embolism.

A right-sided hemiplegia generally appears because the left middle cerebral artery is the usual seat of impaction. This, with a partial loss of sensation, and the general symptoms, as detailed in the summary appended, make up its distinctive features. Erlenmeyer may be quoted as authority for the stated normality of the pupils.

Recovery is usually not long delayed, and the paralysis disappears coincidently with the return of consciousness. A complete absence of paralysis and the occurrence of a variety of epileptiform convulsions have been reported in a few cases.

CEREBRITIS

would not require mention in this connection, but that some exceptional cases are on record, in which coma has been suddenly induced by the rupture of an abscess and escape of its contained pus into the cerebral substance. In these instances slight unilateral convulsions have been noted, and the appearance of the discharge through the auditory, nasal or orbital openings, and the general evidence of inflammatory action, are sufficient diagnostic signs.

SYNCOPE

is so familiar a condition that it is quite unnecessary to enter into its description. Its consideration here is only valuable on account of its relation to other causes of unconsciousness. It is in all cases due to an arrest of function in the cerebral cortex through failure of its arterial blood supply, whether that failure is incident to a general or local anæmia, to sudden failure of the circulation, temporary arrest of the heart's action, or to extensive hemorrhage from any source.

EPILEPSY

it is equally needless to describe in detail. Its symptoms are well-known, and in ordinary cases easily recognized. After the stage of convulsive action has passed, and coma has depended, its recognition may, however, be more difficult.

The formerly livid countenance takes on a pale ashen hue; the pulse becomes feeble and irregular; temperature remains high—in some cases as high as 105° F.—the pupils contract, and frothy saliva, sometimes streaked with blood from the bitten tongue, is found upon the lips.

Paralysis is never proper to epilepsy, but it should be remembered that "a paroxysm of epilepsy may act as an exciting cause of an apoplectic seizure." A case of this kind is reported in a recent number of the New York Medical Record.

CATALEPSY

is the cause of a peculiar form of unconsciousness comparatively easy to differentiate.

Almost invariably peculiar to the female sex; paroxysmal in character; of uncertain duration and constant recurrence; and attended with remarkable muscular rigidity and contortion, it is little apt to offer any possibilities of error.

The feeble, but regular, pulse and respiration; normal temperature; dilated and sensitive pupils; open and tremulous eyelids; anæmic retina, are points worthy of remembrance.

CEREBRAL HYSTERIA, in certain rare cases, is characterized by a sudden loss of consciousness, which may continue for several hours, with slight intervals. It is almost without exception confined to women. It is not accompanied by paralysis, and involves only a partial suspension of intellection, sensation and special sense. The patient can be momentarily aroused, but suffers an immediate relapse. There is little evidence of disturbance of the circulation or respiration. Consciousness is speedily restored on the application of the cold douche.

INSOLATION, or sunstroke, although easy of recognition by means of the presence and prevalence of its exciting cause, is obscure in its pathology. It varies, of course, in duration and intensity. In certain cases cerebral hemorrhage is induced, when paralysis and other symptoms proper to the latter appear.

The pulse always varies; the rapid and somewhat stertorous respiration is sometimes accompanied by a low moaning sound; the temperature ranges from 108° to 110° F.; the skin is peculiarly harsh and hot, and the pupils contracted and insensible. Vomiting and purging are dangerous symptoms.—*Chicago Med. Jour.*

[To be continued in September Number.]

